# Supplementary figures and images for: PD-L1 is expressed on human activated naive effector CD4+ T cells. Regulation by dendritic cells and regulatory CD4+ T cells
Source: PLoS One. 2021 Nov 18;16(11):e0260206. doi: 10.1371/journal.pone.0260206 (PMC8601581; doi:10.1371/journal.pone.0260206)

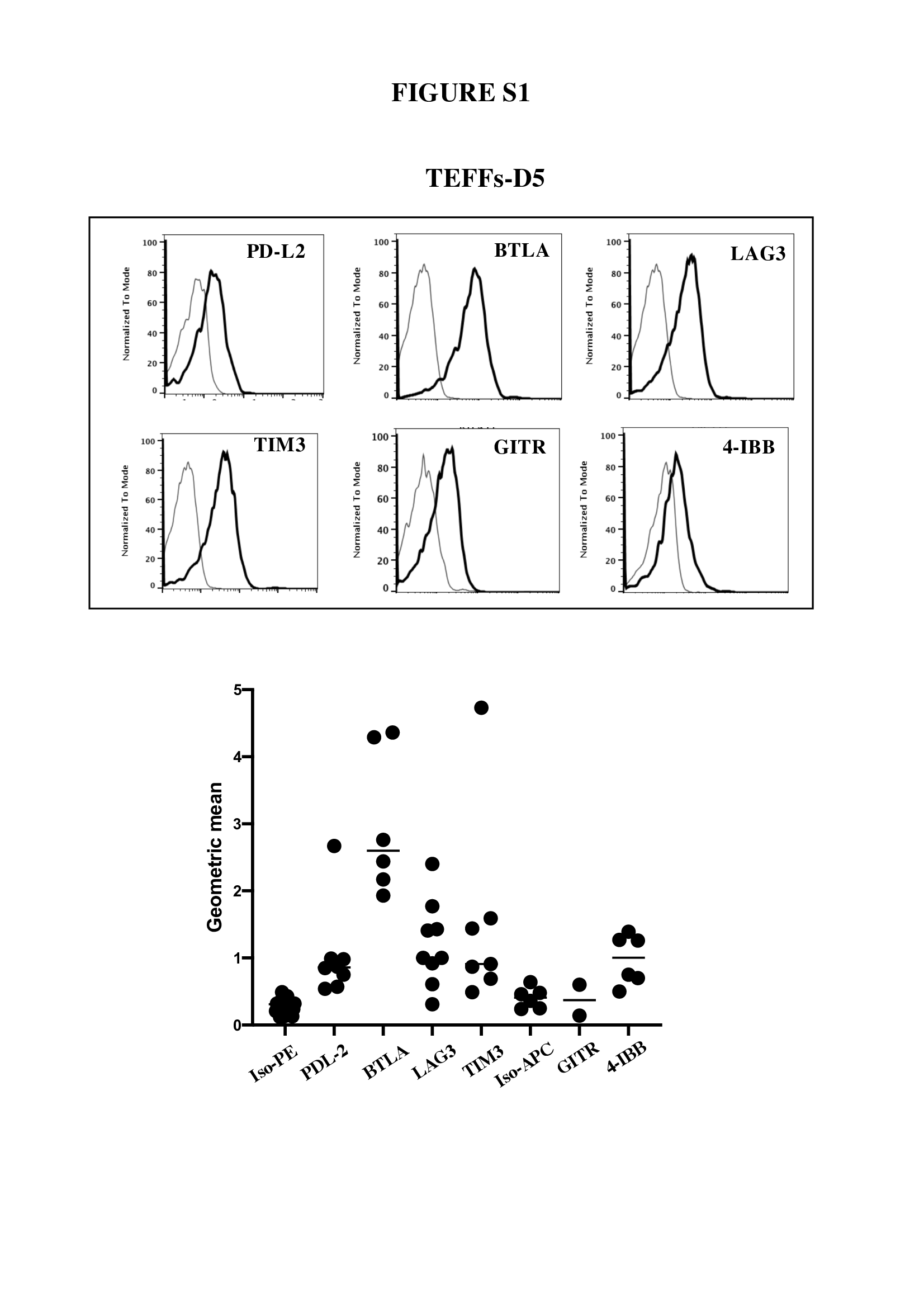

Supplement: S1 Fig — Upper panel: representative histograms of surface receptors on gated, CTV-stained cells on D5 of proliferation assay. For each protein, the specific conjugated isotype control (grey solid line) is shown (Iso-PE- or Iso-APC). Lower panel: graphs of the geometric mean fluorescence intensity of the respective markers in independent experiments (n = 2 to 9). Bars represent the means of all experiments for each proteins. (TIF) [file pone.0260206.s001.tif]

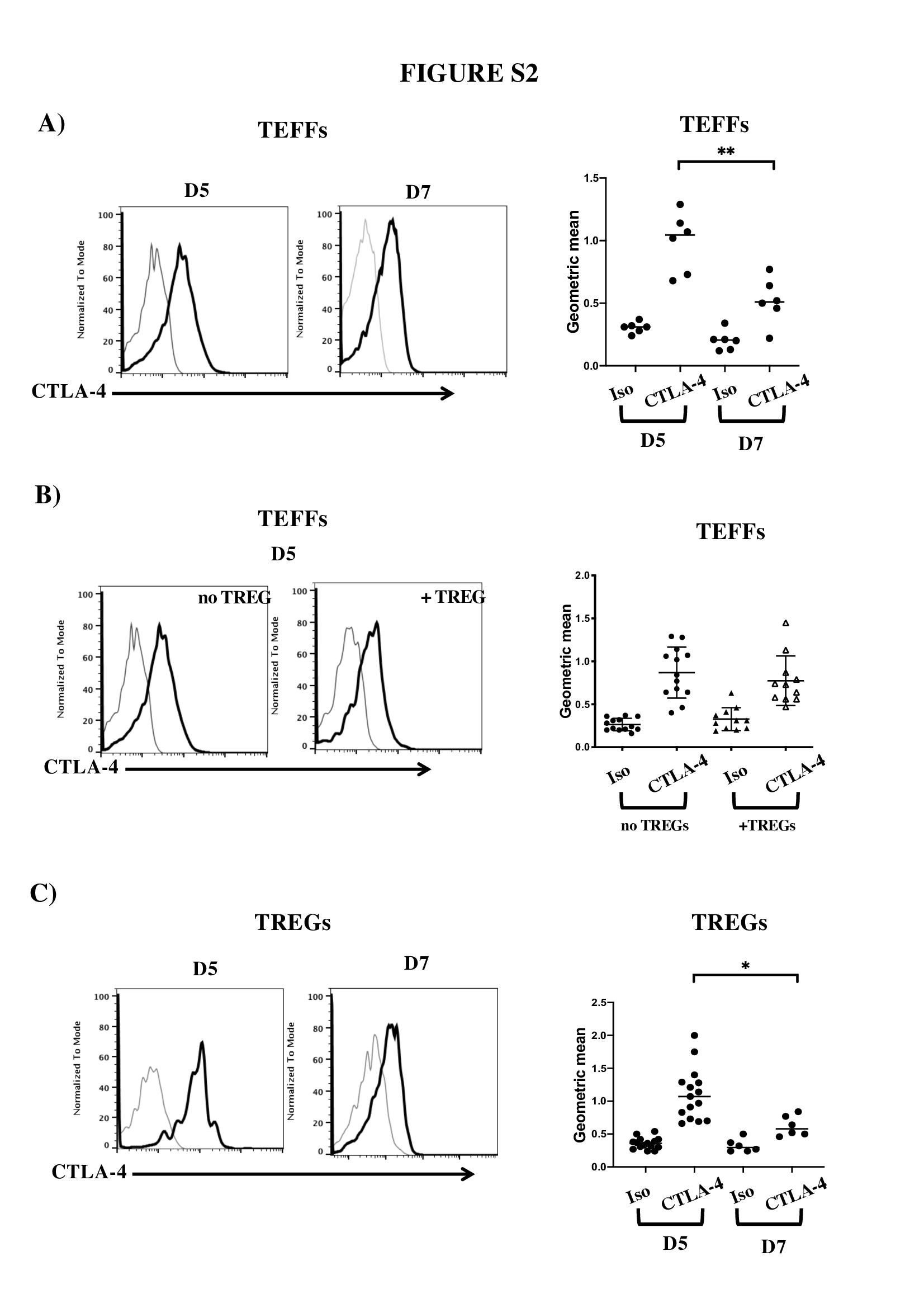

Supplement: S2 Fig — A) Left panel: representative histograms of CTLA-4 expression on gated CTV-stained TEFFs co-cultured with DCs-SEE for 5 days (D5) or 7 days (D7). Right panel: graphs of the geometric mean fluorescence intensity of CTLA-4 expression in independent experiments (n = 6 on D5 and D7). The isotype control (Iso) is shown. B) Left panel: representative histograms of CTLA-4 expression on gated, CTV-stained TEFFs co-cultured with DCs-SEE in the absence (no TREGs) or presence of TREGs (+TREGs) for 5 days (D5). The TEFF:TREG ratio was 2:1. Right panel: graphs of the geometric mean fluorescence of CTLA-4 expression in independent experiments (n = 13 on D5). C) Left panel: representative histograms of CTLA-4 expression on gated, CFSE-stained TREGs co-cultured for 5 days (D5) or 7 days (D7). Right panel: graphs of the geometric mean fluorescence intensity of CTLA-4 expression in independent experiments (n = 15 on D5, n = 6 on D7). * p≤0.05 in a M-W test, ** p≤0.01 in in an M-W test. Bars represent the means of all experiments for each proteins. (TIF) [file pone.0260206.s002.tif]

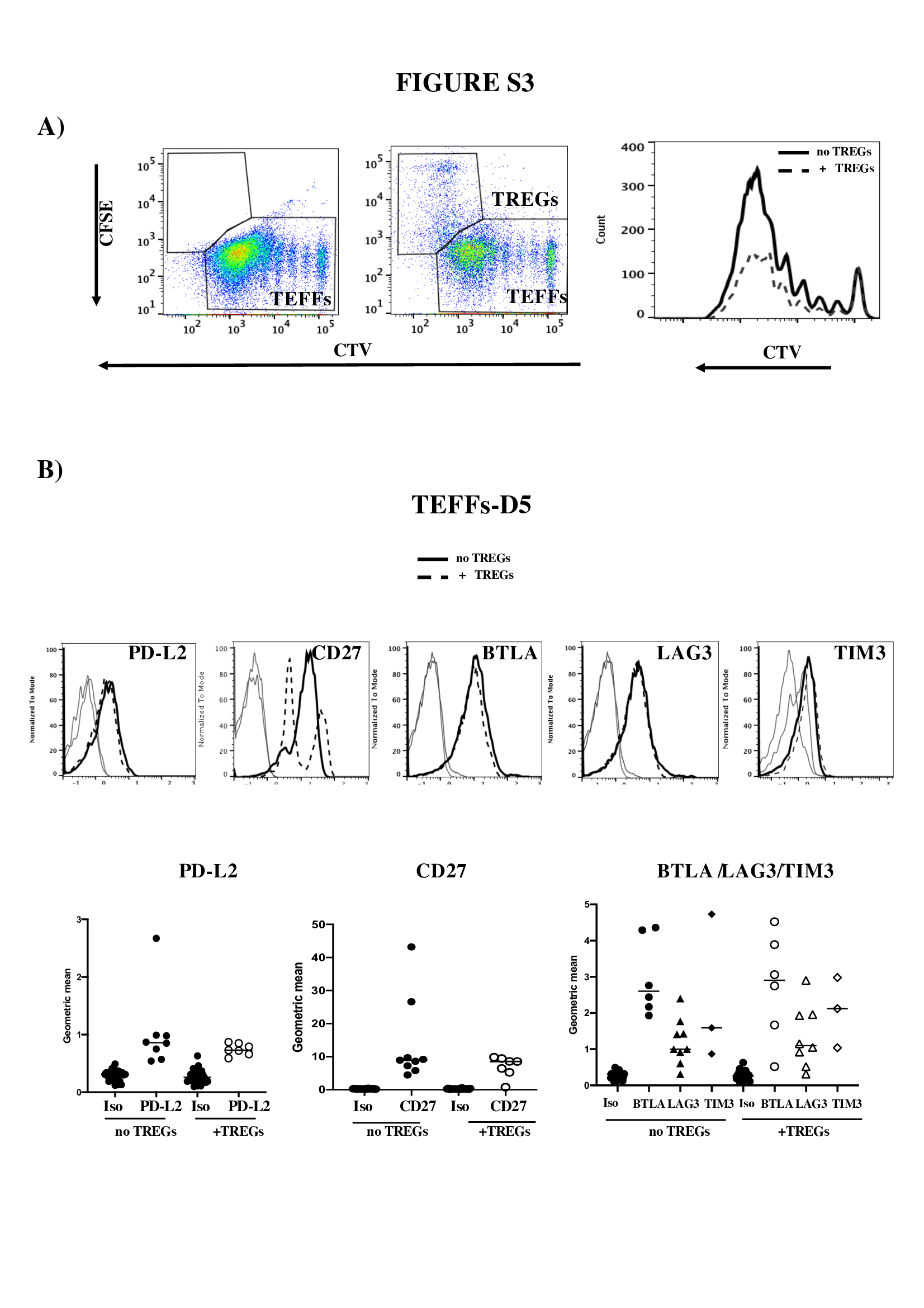

Supplement: S3 Fig — A) Representative flow cytometry dot plots and histograms of the proliferation of CTV-stained TEFFs on D5 of co-culture, in the absence (left dot plot and the solid black line on the histogram) or presence of CFSE-stained TREGs and a TEFF:TREG ratio of 2:1 (right dot-plot and dashed black line on the histogram). B) Representative overlay histograms of different surface receptors in the absence (solid black lines) or presence of TREGs (dashed black line). The isotype control (Iso) is shown for each protein (solid grey lines). Lower panel: graphs of the geometric mean fluorescence intensity of each of the markers, in independent experiments (n = 3 to 8). Bars represent the means of all experiments for each proteins. (TIF) [file pone.0260206.s003.tif]

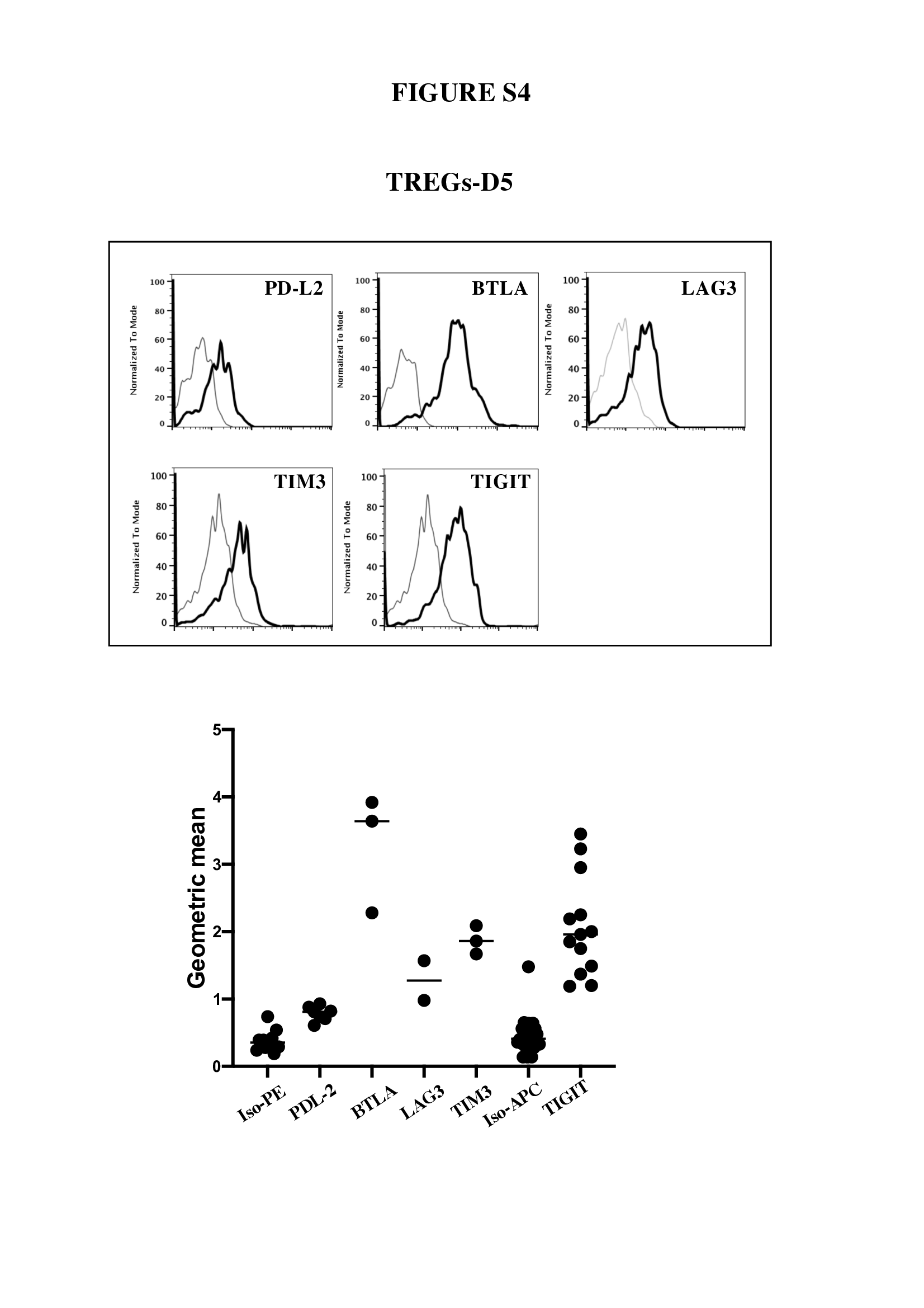

Supplement: S4 Fig — Upper panel: representative histograms of different receptors expressions (solid black lines) on the gated CFSE-stained TREGs on D5 of the co-culture. Lower panel: graphs of each marker’s geometric mean intensity on D5 (n = 2 to 12, depending on the marker and the time course studied). The isotype control (Iso) is shown for each protein (grey lines). Bars represent the means of all experiments for each proteins. (TIF) [file pone.0260206.s004.tif]

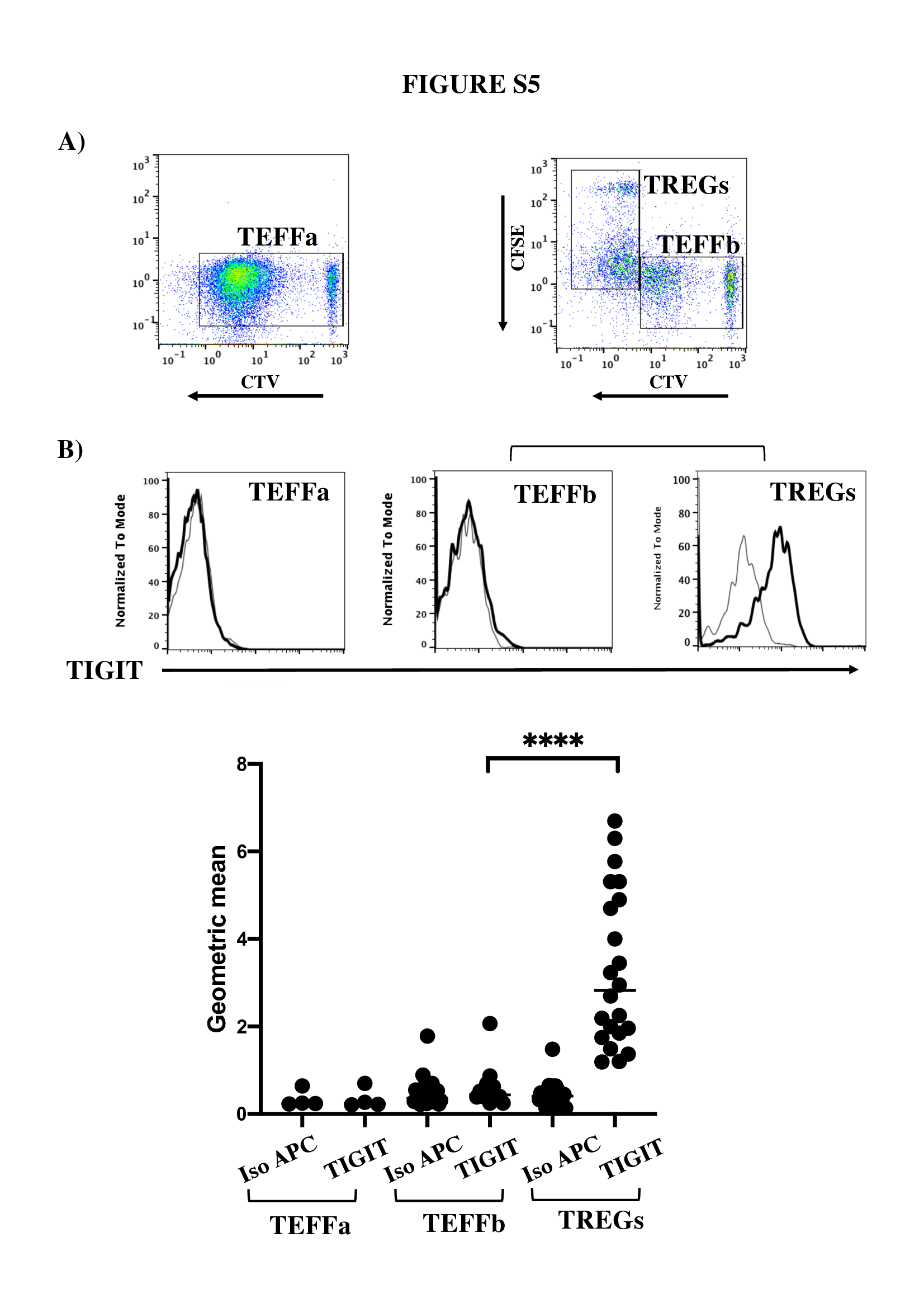

Supplement: S5 Fig — A) Representative flow cytometry dot plots for cell proliferation on D5 of the co-culture. CTV-stained TEFFs were co-cultured with DCs-SEE in the absence (TEFFa) or the presence of CFSE-stained TREGs (TEFFb) with a TEFF:TREG ratio of 2:1. B) Representative histograms of TIGIT expression (solid black lines) on gated CTV-stained TEFFs in the absence of TREGs (TEFFa) or in the presence of TREGs (TEFFb) and on gated CFSE-stained TREG (TREGs). The TEFF:TREG ratio was 2:1. The isotype control (Iso-APC) is shown for each population (grey lines). Lower panel: graphs of the geometric mean fluorescence intensity of TIGIT expression on gated, CTV-stained TEFFs (TEFFa and TEFFb) and on gated, CFSE-stained TREGs in independent experiments (n = 5 to 30, depending on the sub-population studied). **** p≤0.0001 in an M-W test. Bars represent the means of all experiments for each proteins. (TIF) [file pone.0260206.s005.tif]
